# Astaxanthin Complexes to Attenuate Muscle Damage after In Vivo Femoral Ischemia-Reperfusion

**DOI:** 10.3390/md17060354

**Published:** 2019-06-14

**Authors:** Marisol Zuluaga Tamayo, Laurence Choudat, Rachida Aid-Launais, Olivier Thibaudeau, Liliane Louedec, Didier Letourneur, Virginie Gueguen, Anne Meddahi-Pellé, Anne Couvelard, Graciela Pavon-Djavid

**Affiliations:** 1 INSERM U1148, Laboratory for Vascular Translational Science, Cardiovascular Bioengineering, Université Paris 13, Av. Jean-Baptiste Clément 93430 Villetaneuse France/ CHU X. Bichat, 46 rue H. Huchard, 75018 Paris, France; marisolzuluaga@hotmail.com (M.Z.T.); rachida.aid@inserm.fr (R.A.-L.); liliane.louedec@inserm.fr (L.L.); didier.letourneur@inserm.fr (D.L.); virginie.gueguen@univ-paris13.fr (V.G.); anne.pelle@univ-paris13.fr (A.M.-P.); 2 Pathology Department, Bichat Hospital, AP-HP, 46 rue H. Huchard, 75018 Paris, France; laurence.choudat@aphp.fr (L.C.); anne.couvelard@bch.aphp.fr (A.C.); 3 Plateau de Morphologie UMR 1152 Université Paris Diderot, Université de Paris, Bichat Hospital, AP-HP, 46 rue H. Huchard, 75018 Paris, France; olivier.thibaudeau@inserm.fr; 4 Université Paris Diderot, Université de Paris, 16 Rue Henri Huchard, 75018 Paris, France

**Keywords:** astaxanthin, ischemia/reperfusion injury, reactive oxygen species, oxidative stress, cyclodextrin

## Abstract

(1) Background: Reperfusion injury refers to the cell and tissue damage induced, when blood flow is restored after an ischemic period. While reperfusion reestablishes oxygen supply, it generates a high concentration of radicals, resulting in tissue dysfunction and damage. Here, we aimed to challenge and achieve the potential of a delivery system based on astaxanthin, a natural antioxidant, in attenuating the muscle damage in an animal model of femoral hind-limb ischemia and reperfusion. (2) Methods: The antioxidant capacity and non-toxicity of astaxanthin was validated before and after loading into a polysaccharide scaffold. The capacity of astaxanthin to compensate stress damages was also studied after ischemia induced by femoral artery clamping and followed by varied periods of reperfusion. (3) Results: Histological evaluation showed a positive labeling for CD68 and CD163 macrophage markers, indicating a remodeling process. In addition, higher levels of Nrf2 and NQO1 expression in the sham group compared to the antioxidant group could reflect a reduction of the oxidative damage after 15 days of reperfusion. Furthermore, non-significant differences were observed in non-heme iron deposition in both groups, reflecting a cell population susceptible to free radical damage. (4) Conclusions: Our results suggest that the in situ release of an antioxidant molecule could be effective in improving the antioxidant defenses of ischemia/reperfusion (I/R)-damaged muscles.

## 1. Introduction

An ischemic condition results in an imbalance in the oxygen production and consumption in the cells and tissues, generating a constant influx of pro-inflammatory reactions that render tissues vulnerable to microvascular dysfunction [1] and to additional injury [2]. Consequently, an oxidative stress status is generated, characterized by an overconcentration of reactive oxygen species (ROS) [3]. An increase of tissue injury and high inflammatory response [4] have been associated with the reperfusion process due to the endogenous antioxidant defense system’s inability to handle the radical load and restore the affected environment [5,6]. In the case of lower limbs where muscles represent the primary mass of tissue [7], damage triggered by ischemia/reperfusion (I/R) represents the most critical effect of the inflicted injury. According to Gardner et al. [8], antioxidant and anti-inflammatory strategies may be useful to treat lower I/R pathologies. Moreover, animal studies have demonstrated the efficacy of antioxidant therapy in preventing or attenuating the I/R injury [2,9].

Natural carotenoids have shown particular antioxidant capabilities to scavenge ROS and enhance the cell’s ability to prevent oxidative stress [10]. Astaxanthin, a xanthophyll carotenoid, has been studied for its antioxidant and anti-inflammatory properties [11]. Pretreatment with oral astaxanthin supplements has shown to reduce oxidative stress and inflammation in rodents presenting ischemic renal injuries [12], and to reduce apoptosis and autophagy in animals with hepatic ischemia [13]. Furthermore, the oral ingestion of either astaxanthin or vitamin E in myocardial I/R injury for 21 days showed a higher cardioprotection when treated with astaxanthin [14]. Intracerebroventricular injection of astaxanthin revealed a reduction in ischemia-related injury in brain tissue through the inhibition of oxidative stress, reduction of glutamate release, and anti-apoptosis [15]. Apart from these positive results, some drawbacks regarding in vivo stability and bioavailability of astaxanthin have also been reported [16]. A strategy based on the use of carriers for in situ delivery of astaxanthin represents a possible solution to enhance its in vivo effect. We previously showed that hydroxypropyl-β-cyclodextrin-astaxanthin (CD-A) complexes allow the stabilization and preservation of astaxanthin activity. CD-A showed the protection of human endothelial cells under exogenous oxidative stress [17]. In this study, we first challenged the efficacy of a polysaccharide system for the in situ delivery of CD-A. Two natural polysaccharides, pullulan and dextran, were selected to produce scaffolds. This choice was supported by previous works, which showed good biocompatibility, biodegradability, and flexibility of pullulan and dextran in the cardiovascular field, when used as delivery systems [18,19,20,21]. Then, the preservation of antioxidant activity of astaxanthin after being loaded into the scaffolds and the non-toxicity of the system were confirmed. Finally, an animal model of I/R injury was selected as the proof of concept. The occlusion of the femoral artery was chosen, since the superficial femoral and popliteal arteries are continuously affected by ischemic and reperfusion periods [22]. Astaxanthin capacity to compensate muscle damages was determined after 45 min of ischemia induced by femoral artery clamping and followed by varied periods of reperfusion (60 min, 7 or 15 days), as compared with the sham (I/R muscles without antioxidant treatment) and control groups. The gracilis muscle was explanted and histologically evaluated to assess tissue response regarding inflammatory cell infiltration, absence of toxicity, and indirect oxidative stress status.

## 2. Results

### 2.1. P/D/CD-A Scaffold Loading and Antioxidant Activity Evaluation

**Figure 1:**
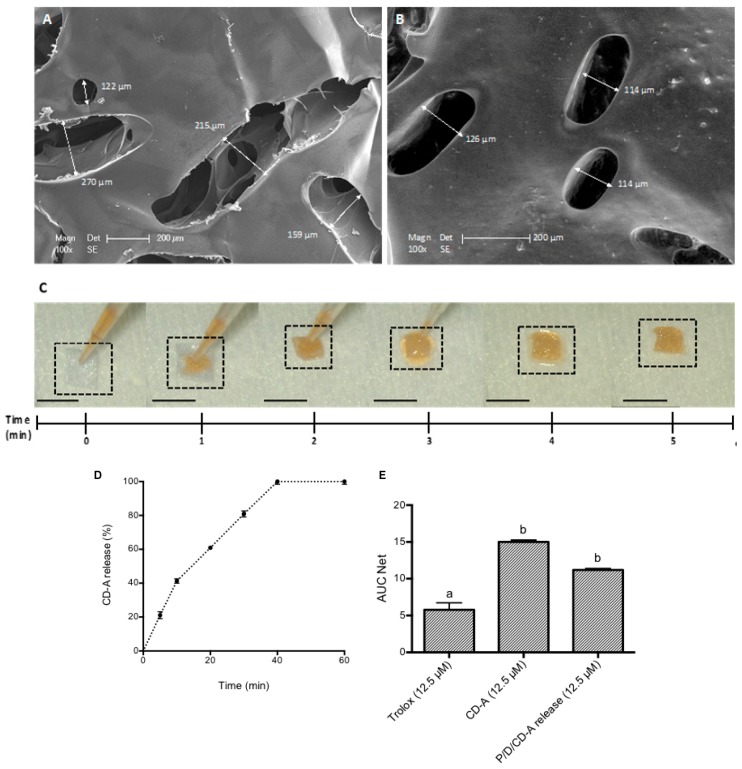
Environmental scanning electron microscopy (ESEM) of the (**A**) pullulan/dextran (P/D) scaffold and (**B**) P/D/hydroxypropyl-β-cyclodextrin-astaxanthin (CD-A) scaffold. (**C**) P/D scaffolds were loaded with 10 µL of either saline solution or CD-A until complete absorption. (**D**) CD-A release kinetics from the P/D scaffolds during 60 min in PBS (pH 7.4). The results indicate mean ± SD (*n* = 46). (**E**) Antioxidant activity evaluation of CD-A and P/D/CD-A release product at 12.5 µM compared to Trolox at 12.5 µM standard antioxidant; results are presented as area under the curve (AUC) net values; the results are expressed as mean ± SD (*n* = 46); values connected with the same letter (a, b) are not significantly different considering *p* < 0.05 as the significance level.

Environmental scanning electron microscopy (ESEM) images reflected a heterogeneous microporous structure of the pullulan/dextran (P/D)-based scaffold (Figure 1A,B), spread all over the surface. Interestingly, the addition of the CD-A within the P/D scaffold smoothed the scaffold pores (Figure 1B). As presented in Figure 1C, the freeze-drying process had an easy handle to the scaffold, as well as a high and fast molecule-loading capacity. Five minutes were required for the complete absorption of the molecule (10 µL/scaffold). The volume needed to charge the scaffold was determined as the swollen maximum capacity without volume saturation (data not shown). The initial dimension of the scaffold was reduced after the complete absorption of different conditions, varying from 5 × 5 mm to 3 × 3 mm. 

P/D/CD-A release product was quantified regarding its concentration and antioxidant capacity. First, a calibration curve using different concentrations of CD-A (0–12.5 µM astaxanthin concentration) versus absorbance at 472 nm was plotted (Figure S1A), then the P/D scaffold was charged with the maximum amount used for the CD-A quantification (2.5 mg; astaxanthin concentration 12.5 µM) and P/D/CD-A controlled kinetics release was reported. Figure 1D indicates a fast release during the first 20 min (50%) and a total release after 60 min. Then, calibration curves using Trolox (standard antioxidant, 0–50 µM) and CD-A (0–50 µM) were plotted versus the area under the curve (AUC) net (Figure S1B). Slope ratio from both curves indicated a high antioxidant capacity of CD-A when compared to the standard antioxidant (2.8 mM Trolox Equivalent Antioxidant Capacity (TEAC)). Figure 1E shows a lower AUC net value for CD-A release product (11.2) against 12.7, the expected value for a total CD-A release; thus, indicating a slight retention of the CD-A antioxidant activity by the P/D scaffold. However, when analyzing all antioxidants at the same concentration, CD-A alone or as a release product, both presented a higher antioxidant activity than Trolox, the reference antioxidant molecule. 

### 2.2. Evaluation of In Vitro and In Vivo Toxicity of P/D/CD-A Scaffold

**Figure 2:**
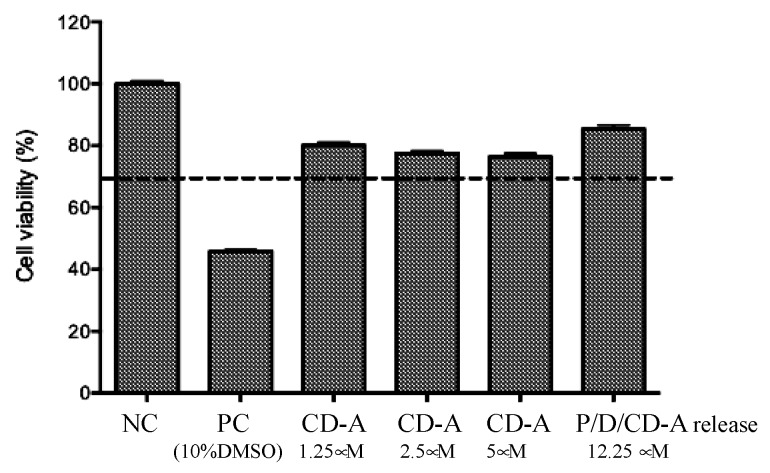
Cell viability assay of CD-A and P/D/CD-A release product by MTT assay. Antioxidant samples were significantly different compared to the positive toxicity control (PC). The results indicate mean ± SD (*n* = 3).

P/D-based scaffolds were autoclaved by UV light and evaluated to determine any sign of in vitro and in vivo toxicity. The toxicity of CD-A and P/D/CD-A release product (12.5 µM) was evaluated after contact with the 3T3 cells for 24 h. This cell line allows the evaluation of toxicity of samples thanks to its high sensitivity. Figure 2 shows an acceptable cell viability (superior to 70% of negative control (NC)), when 3T3 cells were incubated with CD-A (0–5 µM) or with CD-A release products. 

In vivo toxicity was evaluated by monitoring the response of vital organs distant from the implant. No noticeable systemic reaction was triggered by the presence of either the P/D scaffold or the P/D/CD-A antioxidant in the kidney and liver (Figure S2) of the animals subjected to ischemia (45 min) and reperfusion (7 or 15 days) compared to the control group. 

### 2.3. In Vivo Evaluation of P/D/CD-A Scaffolds in Femoral I/R Model

#### 2.3.1. Histological and Immunohistological Analysis

**Figure 3:**
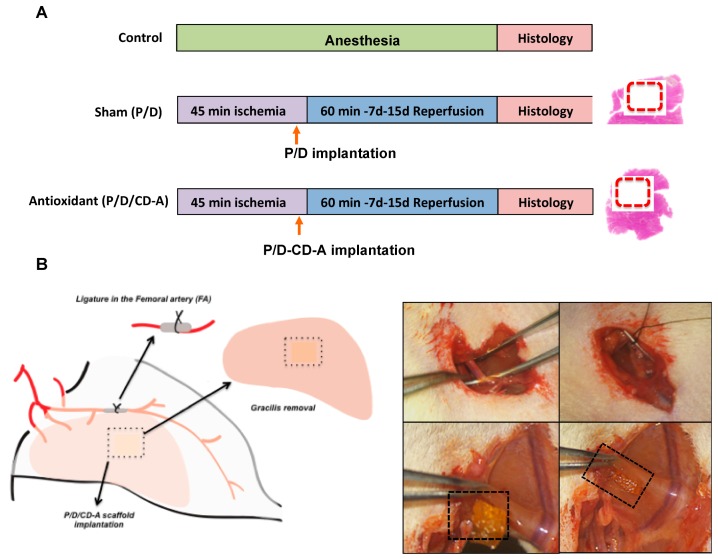
In vivo experimental design and surgical ischemia/reperfusion (I/R) procedure. (**A**) The study animals were assigned into three different groups: control (*n* = 12), sham (*n* = 12), and antioxidant (*n* = 12). (**B**) Control animals underwent 2 h of general anesthesia. The sham and the antioxidant groups underwent 45 min of ischemia induced by the ligature of the femoral artery. Then an intramuscular incision, perpendicular to the muscle fibers, was made in the gracilis muscle, allowing the implantation and keeping hold of the P/D scaffold charged with saline solution in the sham group or the P/D scaffold loaded with the antioxidant molecule (P/D/CD-A) in the antioxidant group, before artery reperfusion for either 60 min, 7 or 15 days. Finally, the gracilis muscles were explanted and histologically evaluated.

During the in vivo implantation study (Figure 3), animals did not present any alteration in their normal behavior or any dietary changes that would reflect suffering. No sign of motion restriction or external acute inflammation was observed when the entire I/R protocol was being followed. After 45 min of ischemia, artery blood flow was gradually restored in the sham and antioxidant groups (confirmed by patency test) in different reperfusion periods (60 min, 7 or 15 days). P/D scaffolds remained in the site of implantation in both P/D (sham) and P/D/CD-A (antioxidant) groups, even after 15 days of analysis, allowing the delivery of the astaxanthin molecule from the scaffold to the surrounded I/R area. 

**Figure 4:**
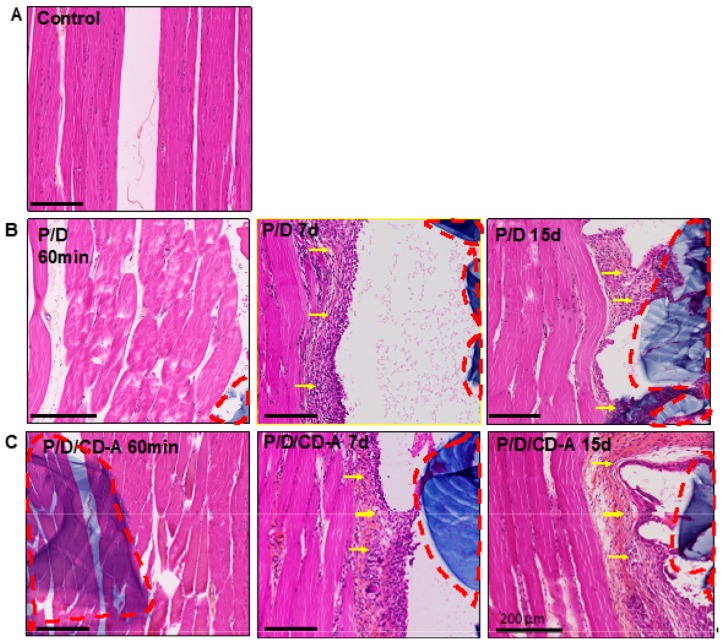
Hematoxylin eosin staining of gracilis muscle of (**A**) control, (**B**) sham, and (**C**) antioxidant groups. The sham and the antioxidant groups that underwent 45 min of occlusion of the femoral artery, were followed by 60 min, 7- or 15-day reperfusions, and treated with P/D scaffold charged with saline solution (sham group) or CD-A (antioxidant group). Scaffolds are stained in blue and surrounded by red dashed lines; gracilis muscle are stained in pink; the fibrous capsule is indicated by yellow arrows. Scale bars, 200 µm; magnification 10×. Experiences were realized in three independents experiments (*n* = 4 animals per group).

Hematoxylin eosin staining (Figure 4) of the control muscles showed a normal morphology with the grouped fibers in a fascicular pattern (Figure 4A). Conversely, P/D and P/D/CD-A groups that were subjected to I/R injury presented muscle fibers with a mild hypertrophic aspect, particularly after 7 and 15 days of reperfusion compared to the control fibers (Figure 4B,C). A fibrous capsule (yellow arrows) that surrounded all P/D-based scaffold-tissue interfaces after 7 and 15 days of reperfusion, was also observed. The thickness of this fibrous capsule was similar in both P/D and P/D/CD-A groups, subjected to the same reperfusion periods.

#### 2.3.2. Phagocyte Responses to Scaffold

**Figure 5:**
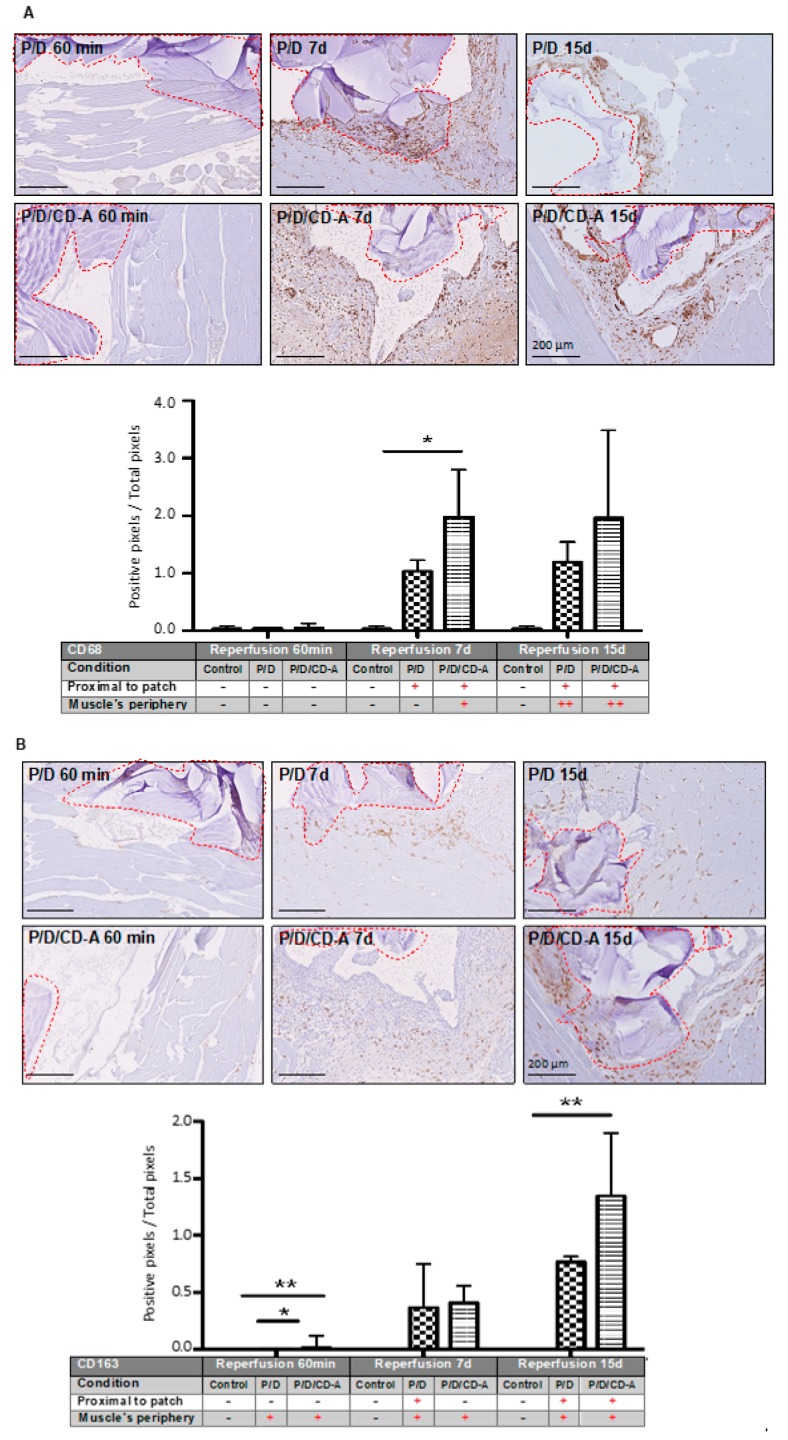
Immunohistochemical detection of monocyte/macrophage populations by CD68 and CD163 markers. Immunological staining and quantification of gracilis muscles containing P/D implanted or P/D/CD-A scaffolds using (**A**) CD68 and (**B**) CD163 markers. In all the cases, the muscle was subjected to 45 min ischemia and reperfused during 60 min, 7 or 15 days. The quantification was done using Matlab and contrasted with the pathologist analysis. The results indicate mean ± SD (*n* = 6). * *p* < 0.05 P/D samples versus the control group and ** *p* < 0.05 PD/CD-A samples versus the control group used to define statistical significance. Red lines indicate the scaffold localization. Scale bars: 200 µm/10× magnification. Experiences were realized in three independent experiments (4 animals per group).

Monocytes/macrophages are phagocytic cells consisting of two cellular subtypes: (i) M1 related to the inflammatory process and positively labeled with the anti-CD68 antibody; (ii) M2 related to the tissue regeneration process and showing positive labeling with anti-CD68 and anti-CD163 antibodies. Evaluation after 60 min of reperfusion demonstrated no significant inflammatory response given by positive CD68 or CD163 markers in any of the I/R groups. An example of histological images is presented in Figure 5A for CD68 positive detection. A significant difference (*p* < 0.05) was found in the total CD68 monocyte/macrophage positive population at 7 days of reperfusion between the P/D and the antioxidant-loaded scaffold P/D/CD-A (Figure 5A, Figure S4A), being almost two times higher according to the image quantification. After 15 days of reperfusion, a non-significant difference was achieved between I/R groups by a slight increase in the P/D CD68 positivity. Few cases of P/D/CD-A scaffolds presented cell colonization composed of CD68 positive marked cells, observed after 15 days of implantation. CD163 positive cells were observed surrounding the scaffold, as well as the gracilis muscle’s periphery, where the scaffold was present (Figure 5B, Figure S4A). After 60 min, a significant difference (*p* < 0.05) was observed between control, P/D, and PD/CD-A samples despite the lower values registered for all three samples. Compared to the sham group, there were no significant changes in the number of cells with positive staining for the CD163 marker after 7 days of reperfusion in the P/D/CD-A group. However, a significant increase of M2 repair macrophages (*p* < 0.05) was observed in the P/D/CD-A group after 15 days. 

#### 2.3.3. Nrf2/HO1/NQO1 Endogenous Antioxidant Systems

**Figure 6:**
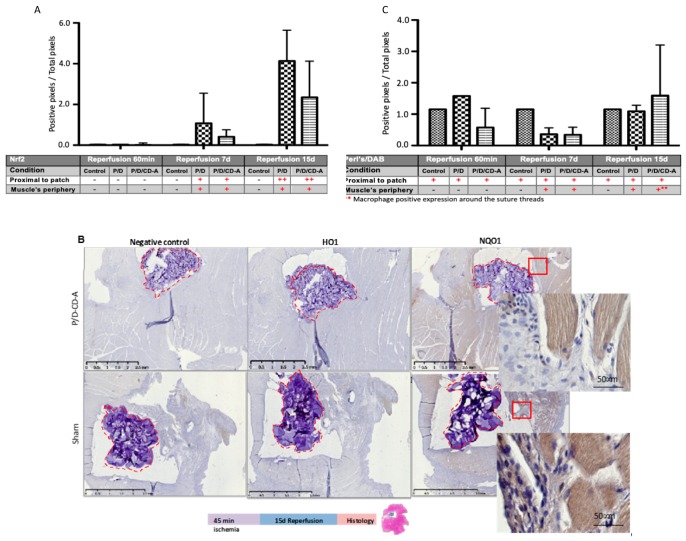
Nuclear factor-erythroid 2-related factor 2 (Nrf2), NAD (P) H: quinone oxidoreductase 1 (NQO1), heme oxygenase-1 (HO1) and Perls/DAB (3,3′-diaminobenzidine) detection in the gracilis muscle loaded with P/D or P/D/CD-A scaffolds at different reperfusion periods. Immunohistochemical detection and quantification of (**A**) Nrf2 (**B**) NQO1, HO1, and (**C**) Perls/DAB, which were evaluated for positive retention of iron in the proximal region of the scaffold by the red blood cells. The quantification of Nrf2 and Perls/DAB was done using Matlab and contrasted with the pathologist’s analysis. The results indicate mean ± SD (*n* = 6). * *p* < 0.05 P/D samples versus the control group and ** *p* < 0.05 PD/CD-A samples versus the control group used to define statistical significance. Red lines indicate the scaffold localization. Scale bars: 200 µm/10× magnification. Experiences were realized in three independent experiments (4 animals per group).

The effect of CD-A local treatment on nuclear factor-erythroid 2-related factor 2 (Nrf2) translocation in the gracilis muscle was studied under I/R conditions. Nrf2 is a crucial transcription factor, which regulates antioxidant defense comprising antioxidant enzymes heme oxygenase-1 (HO1) and NAD (P) H: quinone oxidoreductase 1 (NQO1) [23]. Regarding this, the expression of NQO1 and HO1 proteins was also evaluated. The immunohistochemical detection showed a positive phosphorylated Nrf2 nuclear staining at 7 and 15 days of reperfusion in the P/D/CD-A group (Figure 6A; antibody positive control in a rat and mouse heart is presented in the supplementary data, Figure S3). The quantitative analysis reflected a positive staining which gradually went along with the increased reperfusion periods in both groups (Figure 6A, Figure S4B). It is worth noting that even after 15 days, the P/D/CD-A group showed less Nrf2 positivity compared to the sham group wherein Nrf2 expression was more pronounced. Nonetheless, the variability of the responses between both groups did not lead to significant differences. The expression levels of HO1 and NQO1 were evaluated on the gracilis muscle under I/R conditions after 15 days of reperfusion. After this period, we observed no enhancement in HO1 expression by immunohistochemical staining, neither in sham nor in P/D/CD-A groups. However, we observed a lower NQO1 protein labeling in the P/D/CD-A group compared to the sham (Figure 6B).

#### 2.3.4. Detection of Oxidative Stress induced by Iron Overload in the Tissues

A positive tissue staining with Perls/DAB (3,3′-diaminobenzidine) was differentiated from red blood cells, heme or non-heme iron fixation. After 60 min and 7 days of reperfusion, the P/D/CD-A group showed a decrease in the Perls/DAB positive staining compared to the sham group. However, after 15 days of reperfusion, a slight increase in the non-heme iron fixation in the muscle area next to the implanted scaffold in either the sham or the antioxidant group suggested a retention by macrophages. Positive heme iron fixations of the red blood cells were noticed in all the groups (Figure 6C) and a negative expression was found when Perls was not amplified by the DAB component in all conditions (Figure S4C), contrasting with the positive stain observed after DAB staining attributed to the presence of red blood cells (heme iron staining). Nevertheless, any retention in the gracilis muscle or the surrounded scaffold was found using both Perls or DAB techniques independently in all the groups (Figure S2C, Figure S4C). 

## 3. Discussion

As the main results of this study, a polymeric system composed of a mixture of two polysaccharides, pullulan and dextran, might allow the delivery of an antioxidant molecule such as astaxanthin in an easy and controlled manner. This system could be used as an in situ antioxidant treatment modality to enhance the muscle damage induced by I/R injury. 

In this study, the I/R protocol consisting of the femoral artery clamping in the section between the popliteal and the deep femoral artery during 45 min was chosen. This model was described as the most suitable model of chronic mild ischemia and the most representative of the degree of ischemia seen in patients [24]. Under lower limb I/R conditions, the peripheral muscles are subjected to environmental alterations produced by a higher ROS influx which leads to the depression of the inner body defense mechanism, inducing an imbalance between a burst of ROS and the inability of re-oxygenated cells to handle this radical load, leading to cell degeneration process [5], thereby disturbing their redox stability. Moreover, although muscular damages are induced after ischemic periods, re-establishment of circulation after reperfusion represents a major alteration in the morphological structure [25]. A growing body of literature has reported that after 60 min of reperfusion, a muscular lesion is induced [26,27,28]. 

Previous studies have reported the capacity of antioxidants to attenuate oxidative stress after hind-limb I/R injury [29,30,31,32,33]. Particularly, several studies have evaluated the antioxidant effect of astaxanthin in I/R models via oral and intravenous administration pathways [13,34,35,36]; nevertheless, limitations regarding the biodistribution and stability of the molecule once inside the human or animal body must have been considered. In this study, the in situ delivery of astaxanthin to the site of interest in a controlled manner using a biocompatible scaffold system was intended to address these limitations. The polymer network used in this study was formed through phosphoester linkages between pullulan and dextran by sodium trimetaphosphate (STMP) chemical cross-linking, and scaffold porosity was achieved by the addition of sodium chloride [18]. The fact that the implanted scaffolds remained in the site of implantation within the gracilis muscle even after 15 days of implantation confirmed the feasibility of using this system for the in situ delivery of an antioxidant molecule during a specific period of time. These results corroborate those of a previous work that showed the preservation of the scaffold after 30 days of incubation in a simulated physiological environment [18]. 

Further, the biocompatibility of P/D scaffolds has been widely evaluated in vitro and in vivo. No negative side effects have been reported in a wide range of applications in this regard [18,20,21,37], however no data have been announced concerning the toxicity of P/D scaffolds containing the CD-A molecule. The findings presented here support the non-toxicity of the interaction between the polymeric system and the antioxidant molecule evaluated. Moreover, the foreign body reactions to biomaterials such as scaffolds are controlled by macrophage responses. Macrophages are heterogeneous cell populations, subtypes of which are M1 and M2. According to their functionality, M1 acts as a pro-inflammatory and tumorogenic macrophage, and M2 is regarded as the tissue repair macrophage [38,39]. CD68 antigen is expressed in both M1 and M2 populations, representing a classic sign of inflammation [40]; while CD163 is a specific phenotypic marker for M2 macrophage [41]. The histological results revealed a particularly important cellular reaction in the sham and antioxidant groups within the areas surrounding the scaffold. This reaction represents a classic response to implanted biomaterials, and was mainly arisen out of macrophages and giant cells, which play a central role in the tissue response to the presence of foreign bodies [42,43,44,45]. Additionally, a partial colocalization of CD163 surface marker with the zones also positive for CD68 in the 7 and 15 days of reperfusion, accompanied by a cellular positivity spread along the muscle area, could suggest an immunoregulation and constructive tissue remodeling process [40,46,47]. 

One of the most important cytoprotective systems against oxidative stress is the Keap1-Nrf2 complex [48]. Nrf2 is a pivotal transcription factor which regulates the expression of intracellular antioxidant genes, detoxifying enzymes, and several other ROS-neutralizing proteins [49]. Under physiological conditions, Nrf2 remains inactive in the Keap1-Nrf2 complex near the cell membrane. However, upon high ROS exposure, Nrf2 detaches from the Keap1-Nrf2 complex, translocates to the nucleus [50] and triggers the transcription of more than 200 genes including a set of cytoprotective enzymes, such as NQO1, glutathione peroxidase, HO-1, and superoxide dismutase [51]. It is widely expressed in oxygen-consuming organs, such as muscles, heart, blood vessels, liver, kidneys, and brain. Nrf2 translocation promotes cell survival, preserves cellular redox homeostasis, and plays a key role in reducing inflammation [52]. Through its enzymatic activity, NQO1 can prevent electron reduction of quinones, which results in the production of ROS. Indeed, it has been demonstrated that an increase in the NQO1 level is associated with a decrease of susceptibility to oxidative stress, while a mutation in the NQO1 gene increases the susceptibility to oxidative stress [53]. In this study, the immunohistological results suggest an indirect reduction of the oxidative stress response in the antioxidant-treated group, corroborating our previous findings where we showed the capacity of CD-A to indirectly activate the endogenous antioxidant system of human endothelial cells subjected to exogenous oxidative stress by the Nrf2/HO-1/NQO1 pathway. Moreover, our previous findings demonstrated an upregulation of HO-1 and NQO-1 protein expression levels when the cells received the CD-A treatment [17]. Similarly, a protective effect by astaxanthin pretreatment against brain injuries expressed by a significant increase of the Nrf2, HO-1, and NQO1 mRNA expressions in a cerebral ischemia model was also reported by Lei Pan et al. [36]. In a recent study, Shen et al. [51] showed the involvement of Nrf2 in myocardial I/R injury. The authors pointed out that during the acute myocardial infarction, Nrf2 combines with the antioxidant response element (ARE) to respond to the oxidative stress, reduce the cardiomyocyte apoptosis, and protect the normal function of myocardial tissue.

Iron is a crucial component in living organisms. It plays a vital role as a structural component of heme-containing proteins (Fe^2+^) such as hemoglobin, myoglobin, and cytochromes, and as a metal cofactor (non-heme iron, Fe^3+^) present in several enzymes including Fe-S cluster proteins in mitochondria. Iron homeostasis has been associated with oxidative stress and ROS generation [54]. One of the characteristic reactions of I/R is the reduction of non-heme Fe^3+^ and release of Fe^2+^ ions [55], which are able to react with H_2_O_2_ to generate highly reactive hydroxyl radicals and result in cellular injury [56,57]. Owing to its high sensitivity, the Perls/DAB method was used to provide information about non-heme iron deposition regardless of oxidation states in normal and ischemic conditions by revealing cell population susceptible to free radical damage [58,59]. The results obtained here indicates a response to ROS generation, linked to the reperfusion period and to the non-heme ferrous iron production, which is considered to be critically involved in free radical generation. 

Although we believe that an in situ delivery approach must be considered in the use of the astaxanthin molecule for the treatment of I/R-related pathologies like chronic arterial occlusive disease of the lower extremities, we likewise recognize the limitations it imposes. The I/R model, evaluated here, is in accordance with the degree of ischemia seen in the patients [24]. However, by inducing a mild ischemia in a specific segment of the femoral artery, it seems essential to consider the irrigation of collateral arteries which could reduce the ischemic impact on the adjacent muscles such as the gracilis, thereby making it difficult to evidence the oxidative damage induced in the hind-limb and limiting the quantification of the possible treatment reached by means of the antioxidant. Moreover, as reported here, although in vitro results showed a high scavenging activity at 12.5 µM astaxanthin concentration, and in vivo results reflected a preliminary finding suggesting an improvement in the scavenging activity, by the in situ release of CD-A antioxidant molecule in the vicinity of the muscle in which oxidative stress was generated under I/R injury, an extensive evaluation is still required to select the most appropriate dose of astaxanthin in order to maximize the antioxidant effect. 

In conclusion, the release of CD-A antioxidant through a P/D system in an I/R-damaged environment induces a positive immunological response reflected by an increase of the tissue repair macrophage markers and by the indirect modulation of the Nrf2 transcription factor and the expression of antioxidant enzymes susceptible to oxidative stress. Therefore, this study suggests that the local release of astaxanthin by a polysaccharide scaffold could contribute to the decrease of the damages in the muscles inflicted by I/R injury, by increasing the endogenous antioxidant defenses. 

## 4. Materials and Methods

### 4.1. Chemical and Biological Reagents

Natural astaxanthin (purity >97% HPLC, powder, Lot: 5M4707V), Hydroxypropyl-β-cyclodextrin (DS = 0.67), potassium ferrocyanide (BioUltra, >99.5%), 3-(4,5-dimethyl-2-thiazolyl)-2,5-diphenyl-2H-tetrazolium bromide (MTT), trisodium trimetaphosphate (STMP, lot# MKBQ7691V), sodium chloride (NaCl, lot# SZBA0490), and isopropanol (70% in H2O, Ref: 563935) were purchased from Sigma-Aldrich Co. LLC (Saint-Louis, MO, USA). Pullulan (MW 200000) was purchased from Hayashibara Inc., Okayama, Japan. Dextran (MW 500000), with a degree of branching of 5%, was obtained from Leuconostoc mesenteroids (Pharmacia, Uppsala, Sweden). Acetone, methanol, hydrochloric acid, hydrogen peroxide, and chloroform were all purchased form Carlo Erba Reagents S.A.S (Val-de-Reuil, France). EnVision Dako kit (ref K4063 and ref K4010), Dako REAL peroxidase-blocking solution, and Negative Control mouse IgG1 solution were provided by Dako (Dako, Carpinteria, CA, USA and Dako, Glostrup, Denmark). Mouse anti-rat CD68 and CD163 primary antibodies were obtained from Bio-Rad (MCA341A488, Marnes-la-Coquette, France). Anti-phospho-Nrf2 (S40) antibody was purchased from Abcam (ab76026, Paris, France). The water used was double distilled and deionized. Mouse fibroblast cells CCL 163 (Balb/3T3 clone A31) were purchased from ATCC-LGC Standards S.a.r.l. (Molsheim Cedex, France). Minimum essential medium-L-glutamine (MEM), fetal calf serum (FBS), penicillin–streptomycin- amphotericin (PSA), and trypsin/EDTA solution (TE) were purchased from GIBCO (Life technologies, Carlsbad, CA, USA).

### 4.2. Pullulan/Dextran/CycloDextrin-Astaxanthin Scaffold Preparation and Loading

#### 4.2.1. P/D Scaffold Preparation

Polysaccharide-based scaffolds were prepared according to the protocol proposed by Autissier et al. [60]. Briefly, pullulan, dextran, and NaCl (3:1:4) were mixed in 40% water (*w/v*). Then, 10 g of the polysaccharide solution was withdrawn, to which 1 mL of NaOH 10 M was added, and incubated at 50 °C for 20 min. Next, 300 mg of sodium trimetaphosphate (STMP) (30% *w*/*v*) in 1 mL of water was added as a chemical crosslinking agent. Finally, the mixture was poured into a glass mold of 10 cm × 5 cm × 1 mm. The resultant hydrogel was washed extensively in PBS pH 7.4, freeze-dried (Cryotec, Lyophilizer Crios, France), sterilized under UV light, and stored at room temperature until use. 

#### 4.2.2. CD-A Preparation

To facilitate natural astaxanthin loading into the scaffolds, hydroxypropyl-β-cyclodextrin was previously mixed with astaxanthin according to the following preparation method: Natural astaxanthin (A, 1 mg) in acetone/chloroform (*v*/*v* 1:1) solution was mixed with hydroxypropyl-β-cyclodextrin (CD, 250 mg) dissolved in 12.5 mL of 95% methanol and put in an atmosphere of nitrogen. The mixture was sonicated for 5 min at 35 °C (ultrasonic bath bandelin sonorex rx-100-h) and stirred overnight under light protection. The solution was dried under vacuum, recovered with double distilled water, freeze-dried, and stored at −4 °C until use. 

#### 4.2.3. Scaffold Loading and Characterization

Sterilized P/D scaffolds were cut in 5 × 5 mm squares and loaded with either 10 µL of saline solution for the sham group (P/D) or 2.5 mg CD-A reconstituted in 10 µL saline solution (12.5 µM) for the antioxidant group (P/D/CD-A). Five minutes were required for the solutions to be completely absorbed by the scaffolds. P/D and P/D/CD-A scaffolds were characterized by environmental scanning electron microscopy (ESEM) using a Philips XL 30 ESEM-FEG (Eindhoven, The Netherlands) at an accelerating voltage of 15 keV and at a pressure of 3.5 Torr. 

#### 4.2.4. Release Kinetics Evaluation

To determine the release of CD-A complex from the hydrogel, a calibration curve by plotting the CD-A absorbance (OD) at 472 nm against concentration of the complex (0–15 µM) was established (i-controlTM microplate reader software, TECAN Männedorf, Switzerland). The CD-A release from the scaffold complex was determined as follows: P/D/CD-A scaffolds were immersed in PBS 0.1 M, pH 7.4 at 37 °C, to allow the release of CD-A into the solution. Then, 50 μL of the release solutions were collected at 5, 10, 20, 30, 40, and 60 min. The absorbance of P/D/CD-A release products was recorded at 472 nm, and OD values were reported to the percentage of CD-A concentration based on the calibration curve.

#### 4.2.5. CD-A Antioxidant Activity Evaluation

The antioxidant scavenging capacity of CD-A before and after release from the P/D scaffolds was evaluated using the ORAC (oxygen radical absorbance capacity) method [61]. Briefly, P/D scaffolds were previously loaded with CD-A (12.5 Μm) and immersed into the 1 mL PBS solution at 37 °C for 60 min. 

Solutions of fluorescein (4 nM), 2,2′-Azobis(2-methylpropionamidine) dihydrochloride (AAPH) (160 μM, stressor), and Trolox (0–50 μM, antioxidant standard reference) were prepared in PBS. Fluorescein solution (150 μL) was added to each well (96-well microplate), and then 25 μL of the P/D/CD-A scaffold release product, blank (PBS), or Trolox were distributed in the wells, prior to the addition of AAPH (25 μL). Then fluorescence decay was monitored (485/ 528 nm Ex/Em) at 37 °C for 60 min, and data were taken every minute. 

The area under the curve (AUC) of relative fluorescence was calculated. The AUC values for CD-A release products and Trolox were reported to the AUC value calculated for AAPH (stressor), to indicate the AUC net values. ORAC values were expressed as Trolox equivalent in μM (TEAC, μM) and calculated as the slope ratio from curves, AUCnet, versus the concentration of antioxidant and Trolox, respectively (AUCnet = AUCsample − AUCblank).

#### 4.2.6. Cell Viability Assay

Cell viability was assessed using the MTT assay. To this end, mouse fibroblast cells (3T3) were grown in MEM, supplemented with 10% (*v*/*v*) FBS and 1% PSA. Then, the cells were seeded in a 96-well microplate at a cellular density of 1 × 10^4^ cells/well and cultured overnight. After MEM removal, CD-A (1.25 to 5 μM) or P/D/CD-A scaffold release product, MEM (negative control, PC), or 10% DMSO (positive control, NC) were added to the wells. After 24 h incubation in cell culture conditions, the solutions were discarded and the MTT assay was performed. In this regard, the MTT solution (200 μL, 0.5 mg/mL) was added to the cells and incubated for 3 h at 37 °C. Then, 200 μL of isopropanol was added for 20 min. Next, the absorbance was recorded at 570 nm (i-controlTM microplate reader software, TECAN Männedorf, Switzerland). The samples were considered non-toxic if cellular viability was higher than 70% compared to NC (based on the ISO 10993:2009 1-12 regarding the biological evaluation of medical devices). 

#### 4.2.7. Surgical Procedure and Experimental Design

Animal surgeries were performed in accordance with the “principles of laboratory animal care” and were approved by the Animal Ethics Committee of the Bichat Laboratory (N°2011-14/698-0038). Hind-limb I/R surgeries were performed on male rats weighted 250–300 g (Charles River Laboratory, Wilmington, MA, USA) using an I/R model adapted from Luyt et al. [62]. Animals were anaesthetized with intraperitoneal injection of pentobarbital (30 mg/kg) (Centravet, France). They were randomly assigned to three experimental groups: control (*n* = 12), sham (*n* = 12), and antioxidant (*n* = 12) (Figure 2). Control animals underwent 2 h of general anesthesia. The sham and antioxidant groups underwent 45 min of ischemia induced by the ligature of femoral artery with a 6-0 silk suture. Then an intramuscular incision, perpendicular to the muscle fibers, was made in the gracilis muscle, allowing the implantation and keeping the hold of the P/D scaffold charged with 10 µL saline solution in the sham group, or the P/D scaffold loaded with 12.5 μM of the antioxidant molecule (P/D/CD-A) in the antioxidant group, 5 min prior to the artery reperfusion. Then arterial blood flow was restored and reperfusion periods of 60 min, 7 or 15 days (Figure 2) were evaluated. Finally, rats were sacrificed with an overdose of sodium pentobarbital (150 mg/kg) intraperitoneally injected, and then the gracilis muscles were explanted and histologically evaluated. 

#### 4.2.8. Histological and Immunological Evaluation

Animals were sacrificed 1 h, 7 days or 15 days post-surgery. Gracilis muscles were collected and immersed in 4% formaldehyde during 24 h, dehydrated, and embedded in paraffin according to the standard protocols. Sections at 4 μm thickness (HM 355S microtome, Thermo scientific Waltham, MA, USA) were stained with hematoxylin and eosin. Total phagocytes and type-2 macrophages were identified by anti-CD68 and anti-CD163 antibodies labeling respectively (1:30 working dilutions). Anti-phospho Nrf2 (1:100 working dilution) immunomarker was used to collect information about the muscle general oxidative stress status after I/R injury. DAKO EnVision+ System and 3,3′-diaminobenzidine was used as a chromogen. Iron accumulation in healthy and I/R muscles was quantified by Perls reaction amplified by 3,3′-diaminobenzidine (DAB). After antigen retrieval, samples were incubated in a solution (pH 0.5–0.6, 25 °C) containing 1% HCl and 1% potassium ferrocyanide, followed by H_2_O_2_ incubation [63]. All slides were counterstained with hematoxylin, and digital images were obtained and analyzed using Nanozoomer digital pathology software (Hamamatsu, Japan). 

Samples images were analyzed by two independent methods: 

(i) A mathematical quantitative program: A saturation analysis which highlighted the positive stains in the images created by different immunostaining markers using Matlab software (version 8.5, The MathWorks, Inc., Natick, MA, USA). Each image was separated into its components: hue, saturation, and value color bands. Then thresholds were defined to determine the mask for the region of color corresponding to the positively stained cells represented by brown color spots. Total image pixels of each image were used to calculate the percentage of positive staining as a pixel difference.

(ii) A qualitative method: The analysis of the proximal and distal sections of the muscle was performed by a physician/pathologist in an independent and random manner. The tissue response was scored (macrophages, cellular infiltration, giant cells) according to a relative scoring system [64]: – = no observation, + = mild, and ++ = moderate.

#### 4.2.9. Statistical Analysis

All experiments were repeated at least three times to ensure the reproducibility of each test. Results were expressed as the mean ± SD and statistical analyses were done using one-way ANOVA followed by Tukey’s HSD (honestly significant difference) post hoc test (JMP Software, Version 9; SAS Institute, Cary, NC, USA). Statistical significance was set at *p*-value < 0.05. 

#### 4.2.10. List of abbreviations

Reactive oxygen species (ROS); ischemia/reperfusion (I/R); hydroxypropyl-β-cyclodextrin (CD); hydroxypropyl-β-cyclodextrin-astaxanthin (CD-A); pullulan/dextran-based scaffold (P/D); pullulan/dextran-based scaffold loaded with hydroxypropyl-β-cyclodextrin-astaxanthin (P/D/CD-A scaffold); oxygen radical absorbance capacity (ORAC); area under the curve (AUC); nuclear factor-erythroid 2-related factor 2 (Nrf2); heme oxygenase-1 (HO1); NAD (P) H: quinone oxidoreductase 1 (NQO1).

## Supplementary Materials

The following are available online at https://www.mdpi.com/1660-3397/17/6/354/s1, Figure S1: Calibration curves of CDA and ORAC test, Figure S2: Hematoxylin-eosin staining of liver and kidney from control rats and rats which underwent 45 min of ischemia and were subjected to 7 or 15 days of perfusion, Figure S3: Immuno-histological staining of rat and mouse heart showing the positivity of the antibody anti-phospho-Nrf2, Figure S4: Quantification of positive staining using Matlab.
