# Supplementary material for: Astaxanthin Complexes to Attenuate Muscle Damage after In Vivo Femoral Ischemia-Reperfusion
Source: Mar Drugs. 2019 Jun 14;17(6):354. doi: 10.3390/md17060354 (PMC6627496; doi:10.3390/md17060354)
Supplement: Supplementary file 1 [file marinedrugs-17-00354-s001.pdf]

Supplementary Material

Astaxanthin Complexes to Attenuate Muscle Damage after In Vivo Femoral Ischemia-Reperfusion

Marisol Zuluaga Tamayo[1], Laurence Choudat[2], Rachida Aid-Launais[1], Olivier Thibaudeau[3], Liliane Louedec[1], Didier Letourneur[1], Virginie Gueguen[1], Anne Meddahi-Pellé[1], Anne Couvelard[2,4] and Graciela Pavon-Djavid[1,*]

[1]INSERM U1148, Laboratory for Vascular Translational Science, Cardiovascular Bioengineering; Université Paris 13, 99 Av. Jean-Baptiste Clément 93430 Villetaneuse France/ CHU X. Bichat, 46 rue H. Huchard, 75018 Paris.

[2]Pathology Department, Bichat Hospital, AP-HP, 46 rue H. Huchard, 75018 Paris.

[3]University Paris Diderot, Sorbonne Paris Cité.

*** Corresponding:** Graciela Pavond-Djavid, graciela.pavon@univ-paris13.fr

Supplementary Figures

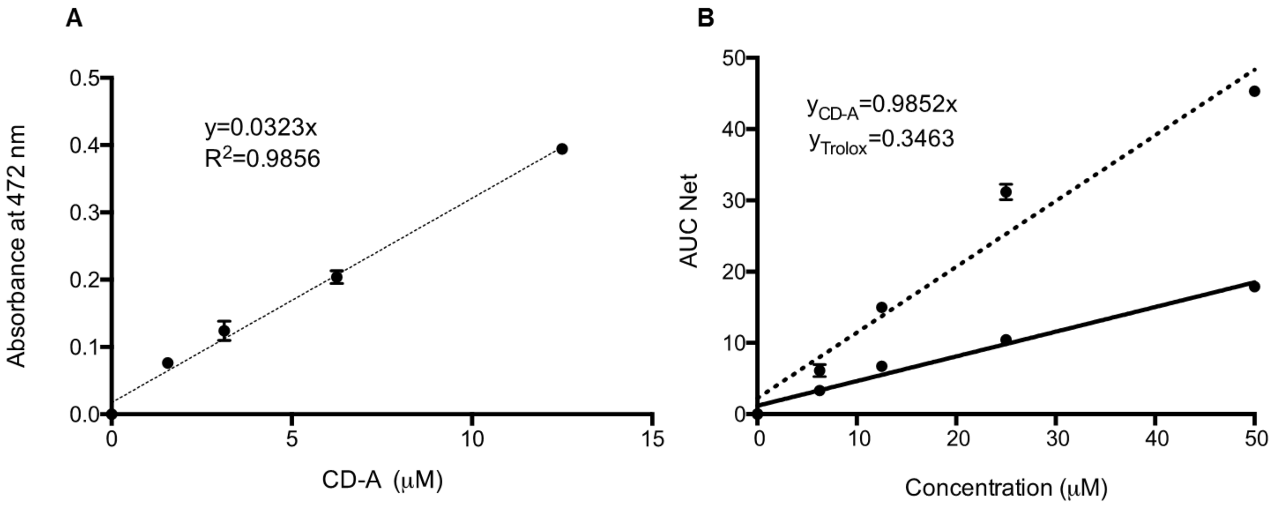

Figure S1: (**A**) Calibration curve of CD-A at concentration ranging from 0-15 μM measure at an absorbance of 472 nm. (**B**) Calibration curve of CD-A and Trolox antioxidant scavenging capacities were analyzed by ORAC method; values are represented as means of AUC net against CD-A or Trolox concentration between 0-50 μM.

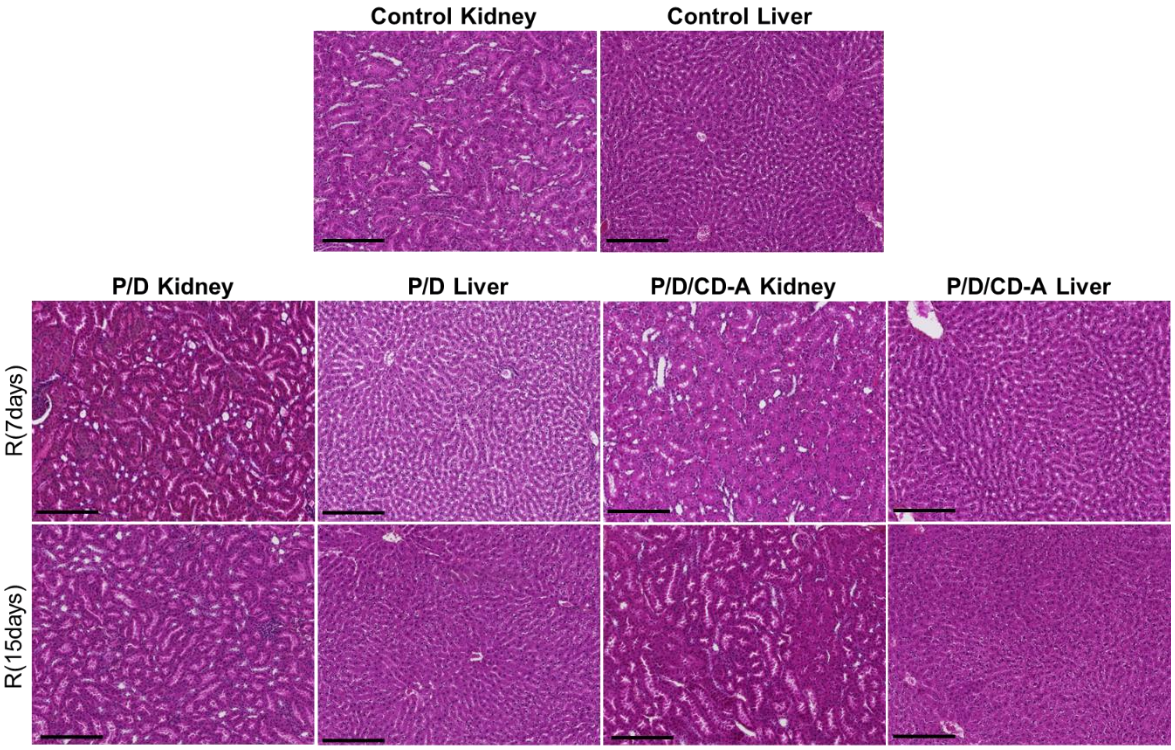

Figure S2: Hematoxylin-eosin staining of liver and kidney from control rats and rats which underwent 45 min of ischemia and were subjected to 7 or 15 days of perfusion. Scale bars, 200 μm; magnification 10×.

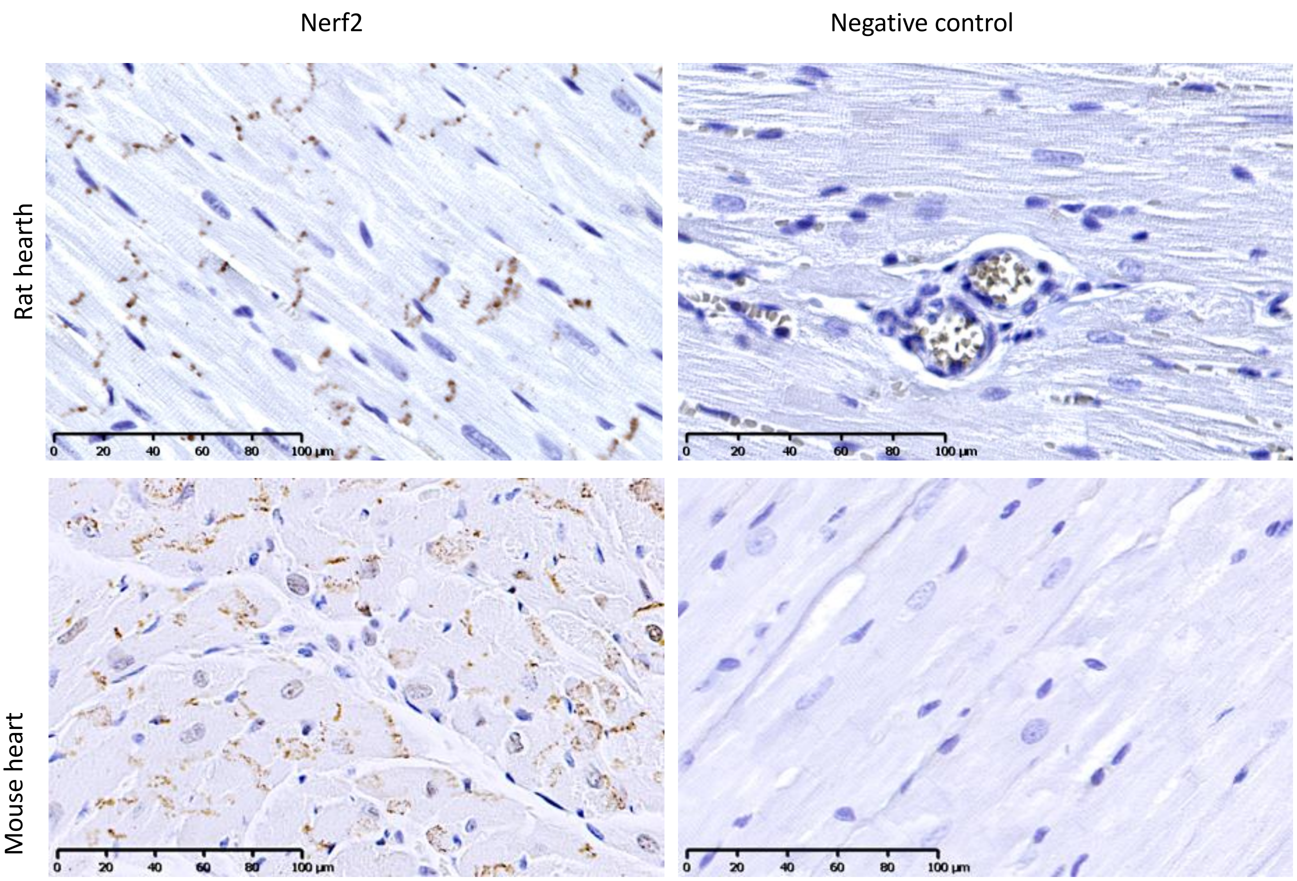

Figure S3: Immuno-histological staining of rat and mouse heart showing the positivity of the antibody anti-phospho-Nrf2.

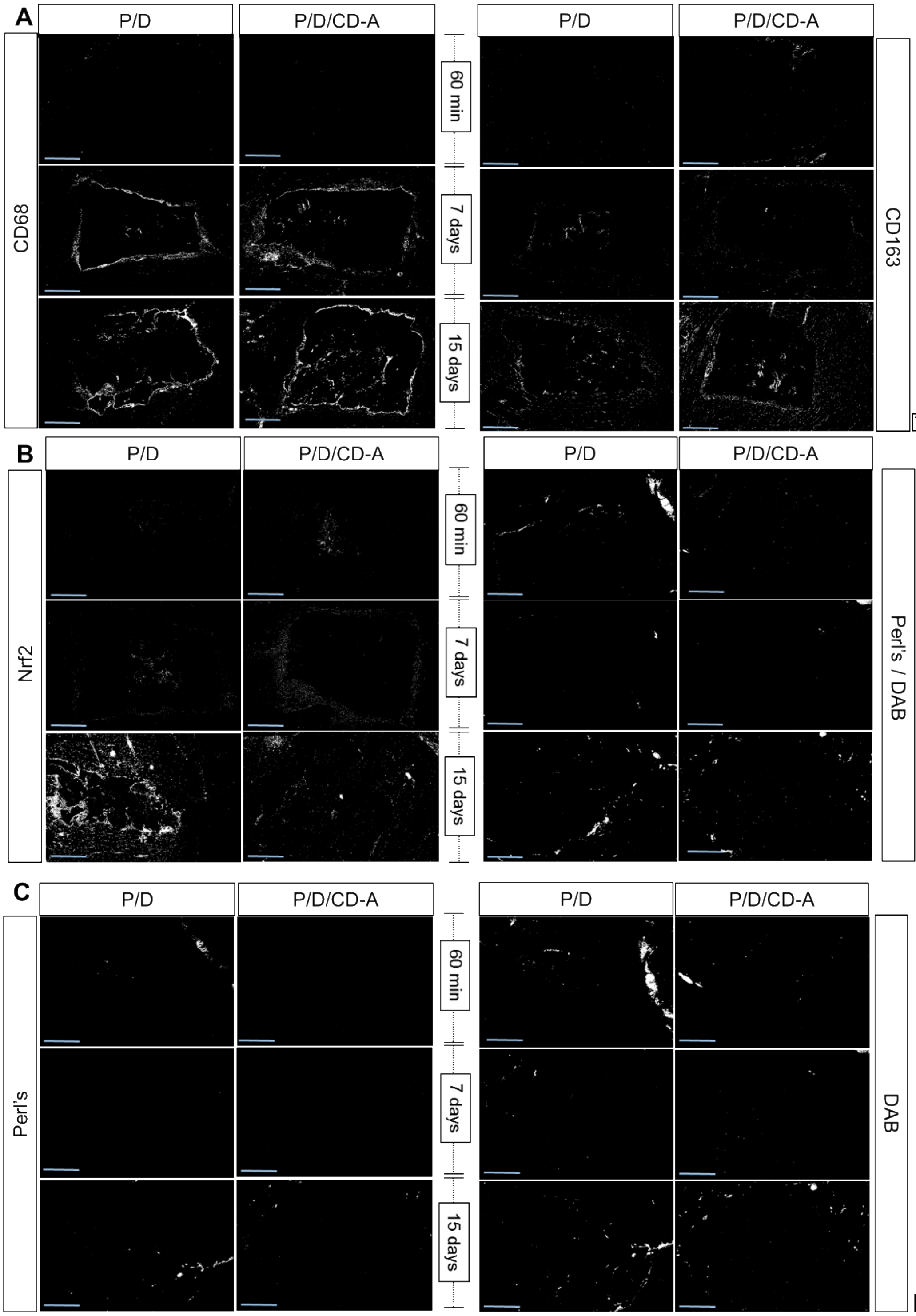

Figure S4: Quantification of positive staining using Matlab. (A) CD68 and CD163 samples, (B) Nrf2 and Perl's/DAB samples, and (C) Perl's and DAB samples. White dots in the images represent the positivity of the respective marker. Scale bars, 2 mm. Images are representing of 4 independent experiments.
